# The role and significance of angiotensin‐converting enzyme 2 peptides in the treatment of coronavirus disease 2019

**DOI:** 10.1002/jcla.23789

**Published:** 2021-05-05

**Authors:** Yang Liu, Huanzhong He, Huilian Huang

**Affiliations:** ^1^ The Department of Anesthesia Huzhou Central Hospital Affiliated Central Hospital HuZhou University Huzhou China; ^2^ Key Laboratory of Molecular Medicine Huzhou Central Hospital Affiliated Central Hospital HuZhou University Huzhou China

**Keywords:** acute respiratory distress syndrome, angiotensin‐converting enzyme 2, coronavirus disease 2019, drug prediction, novel coronavirus

## Abstract

Since the end of 2019, coronavirus disease 2019 (COVID‐19) caused by the novel coronavirus (2019‐nCoV) posed a serious threat to human health and life. Therefore, the discovery of drugs that can effectively prevent and treat COVID‐19 is urgently warranted. In this article, the role and significance of angiotensin‐converting enzyme 2 in drug development and the treatment of COVID‐19 are discussed. It was found that the binding of ACE2 to SARS‐CoV‐2‐RBD involved two core regions (31st and 353rd lysine) and 20 amino acids of the ACE2 protein. The mutation of these amino acids could lead to a great change of the binding ability of ACE2 and SARS‐CoV‐2‐RBD. This information was important for us to find more efficient ACE2 peptides to block the 2019‐nCoV infection. So during this study, we summarized the role of ACE2 in the regulation of 2019‐nCoV infection and stress, and hypothesized that the development and optimization of ACE2 peptide can effectively block 2019‐nCoV infection and reliably treat the COVID‐19.

## Graphical abstract

The binding of ACE2 to SARS‐CoV‐2‐RBD involved two core regions (31st and 353rd lysine) and 20 amino acids of the ACE2 protein. The mutation of these amino acids could lead to a great change of the binding ability of ACE2 and SARS‐CoV‐2‐RBD, so the efficient ACE2 peptides may effectively block the 2019‐nCoV infection.

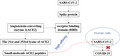

## 1 INTRODUCTION

At the end of December 2019, a newly discovered coronavirus (severe acute respiratory syndrome coronavirus 2 [SARS‐CoV‐2]) rapidly swept across China. The virus causes coronavirus disease 2019 (COVID‐19), leading to severe sequelae, such as acute respiratory distress, septic shock, and multiple organ dysfunction/failure.^1^, ^2^, ^3^ Thus, COVID‐19 poses a serious threat to human life. Since March 2020, the novel coronavirus has spread rapidly worldwide. By March 2021, there have been more than 117.6 million confirmed cases and 2.6 million deaths worldwide, with a mortality rate of >2%. Despite the measures taken worldwide, the virus continues to spread, and the duration of the pandemic remains unknown.

In early case reports, SARS‐CoV‐2 was considered to be less severe than the SARS‐CoV and Middle East respiratory syndrome coronavirus (MERS‐CoV) that caused outbreaks in 2003 and 2012, respectively.^4^, ^5^, ^6^ However, based on the extensive transmission of the virus and the rapid progression of the disease, increasing evidence shows that SARS‐CoV‐2 is more infectious and harmful,^7^ and its impact on human health and the world economy is far greater than that of SARS‐CoV and MERS‐CoV. In the face of the sudden disaster, scientists worldwide strive to develop vaccines and specific drugs for the treatment of this disease. Thus far, several vaccines against COVID‐19 have been approved and utilized. Despite the occurrence of some adverse events, the effectiveness of the vaccines has been affirmed. However, investigators have not yet discovered an effective and specific pharmacological agent for the treatment of COVID‐19. Hence, the battle against SARS‐CoV‐2 remains at the stage of continuous exploration.

## 2 ANGIOTENSIN‐CONVERTING ENZYME 2 (ACE2) AND SARS‐CoV‐2

Since the outbreak of SARS‐CoV‐2, scientists have carried out intensive research to establish a more comprehensive scientific basis for the treatment of COVID‐19. Similar to the pathogenic mechanism of SARS‐CoV, SARS‐CoV‐2 also attaches to the host cell through binding of the receptor binding domain (RBD) in the S1 subunit of S (Spike) protein to the cellular receptor ACE2 and enters the cells via endocytosis.^8^, ^9^, ^10^, ^11^ This leads to progression and deterioration of the disease. SARS‐CoV‐2 can easily invade the human body through the combination of RBD and ACE2. Researchers determined the structure of the SARS‐CoV‐2 spike protein using cryo‐electron microscopy, and found that the SARS‐CoV‐2 spike protein exhibits significantly higher ACE2‐binding affinity than the SARS‐CoV spike protein (10–20‐fold higher).^12^ This fully explains the markedly more efficient transmission of SARS‐CoV‐2 versus SARS‐CoV, and also reveals the important role of ACE2 in the process of SARS‐CoV‐2 infection. Therefore, using ACE2 receptor protein as a target, we may be able to discover a specific drug for the treatment of COVID‐19.

## 3 THE BIOLOGICAL FUNCTIONS OF ACE2

ACE2 is a multifunctional type I transmembrane glycoprotein with a single extracellular catalytic domain.^13^ It belongs to the ACE family and is the homolog of ACE. ACE and ACE2 are involved in the regulation of the renin‐angiotensin system (RAS). The ACE/angiotensin II/angiotensin II type 1 receptor (ACE/Ang II/AT1R) axis regulates signaling pathways to increase blood pressure and promote inflammatory reactions. The signaling pathway regulated by the ACE2/Ang1–7/MAS axis has been associated with vasodilatory, anti‐oxidative, and anti‐inflammatory effects.^14^, ^15^, ^16^ A previous study demonstrated that SARS‐CoV‐2 infections caused a downregulation of ACE2 expression.^17^, ^18^, ^19^ This led to imbalance in the RAS system, promotion of inflammation, and further disease progression. In some patients with cardiovascular disease, diabetes, etc., SARS‐CoV‐2 infection is accelerated by overexpression of ACE2.^20^, ^21^ Consequently, elderly individuals accounted for 80% of the clinical deaths caused by SARS‐CoV‐2, and 75% of those patients suffered from cardiovascular disease, diabetes, and other underlying diseases.

Using single‐cell analysis technology, researchers found that ACE2 is abundantly expressed in human alveolar type II epithelial cells and colonic epithelial cells. Furthermore, gene functional enrichment analysis revealed the genes that are relevant to viral infections in human alveolar type II epithelial cells and colonic epithelial cells with high expression of ACE2. These mainly include genes regulating virus invasion and release, recognizing virus nucleic acid and type I/ III interferon signaling, pro‐inflammatory cytokines and chemokines, the major histocompatibility complex I (MHC I) antigen presentation pathway, and cytokine receptors.^22^, ^23^ The results of that study indicated that entry of the virus into cells through ACE2 leads to undetermined consequences.

ACE2 is an important factor in SARS‐CoV‐2 infection and COVID‐19 progression. Effective blockage of the combination of SARS‐CoV‐2‐RBD and ACE2 is the key to the treatment of COVID‐19. Studies have found that the binding of the ACE2 extracellular domain (18–615 aa) to the immunoglobulin (Ig) FC (ACE2‐Fc) can block the entry of SARS‐CoV‐2. In addition, the symptoms of acute respiratory distress syndrome can be improved by compensating for the decrease in the levels of ACE2 in the lungs during infection through supplementation.^24^ Other studies have found that recombinant ACE2‐Ig (1–709 aa) can effectively prevent the entry of the SARS‐CoV‐2 and SARS‐CoV spike proteins into cells.^25^ As important members of the ACE/Ang II/AT1R axis and ACE2/Ang1–7/MAS axis, ACE2 and ACE participate in important physiological processes in vivo, such as blood pressure regulation, fluid balance, inflammation, cell proliferation, hypertrophy, and fibrosis. In addition, ACE2 plays an important role in regulating cardiovascular function, renal function, and fertility. Therefore, the soluble ACE2‐Fc protein binding to the ACE2 extracellular domain can be used as a drug for the treatment of COVID‐19. In view of the important function of ACE2, it is worth investigating whether the overexpression of ACE2 protein will lead to imbalance of the RAS system and further aggravate the disease.

## 4 THE BINDING SITES OF ACE2 TO SARS‐CoV‐2

It has been found that the new small‐molecule peptides prepared from the N‐terminal 21–43 amino acid protein of ACE2 can effectively bind to SARS‐CoV‐2‐RBD.^26^ However, recent studies using the deep parsing approach for the structural investigation of SARS‐CoV‐2 and ACE2 binding showed that the binding of ACE2 to SARS‐CoV‐2‐RBD involves two core regions (31st and 353rd lysine) of the ACE2 protein.^27^, ^28^ Whether the new small‐molecule peptides prepared from the N‐terminal 21–43 amino acid protein of ACE2 can effectively prevent the binding of the SARS‐CoV‐2 spike protein to ACE2 remains to be verified.

Recently, numerous studies have shown that ACE2 has an important impact on SARS‐CoV‐2 infection and disease prognosis. As a key target for the treatment of COVID‐19, ACE2 has important research value. Research studies are warranted to determine the approach through which the key functions of ACE2 can be used to effectively design molecular targeted agents.

**FIGURE 1:**
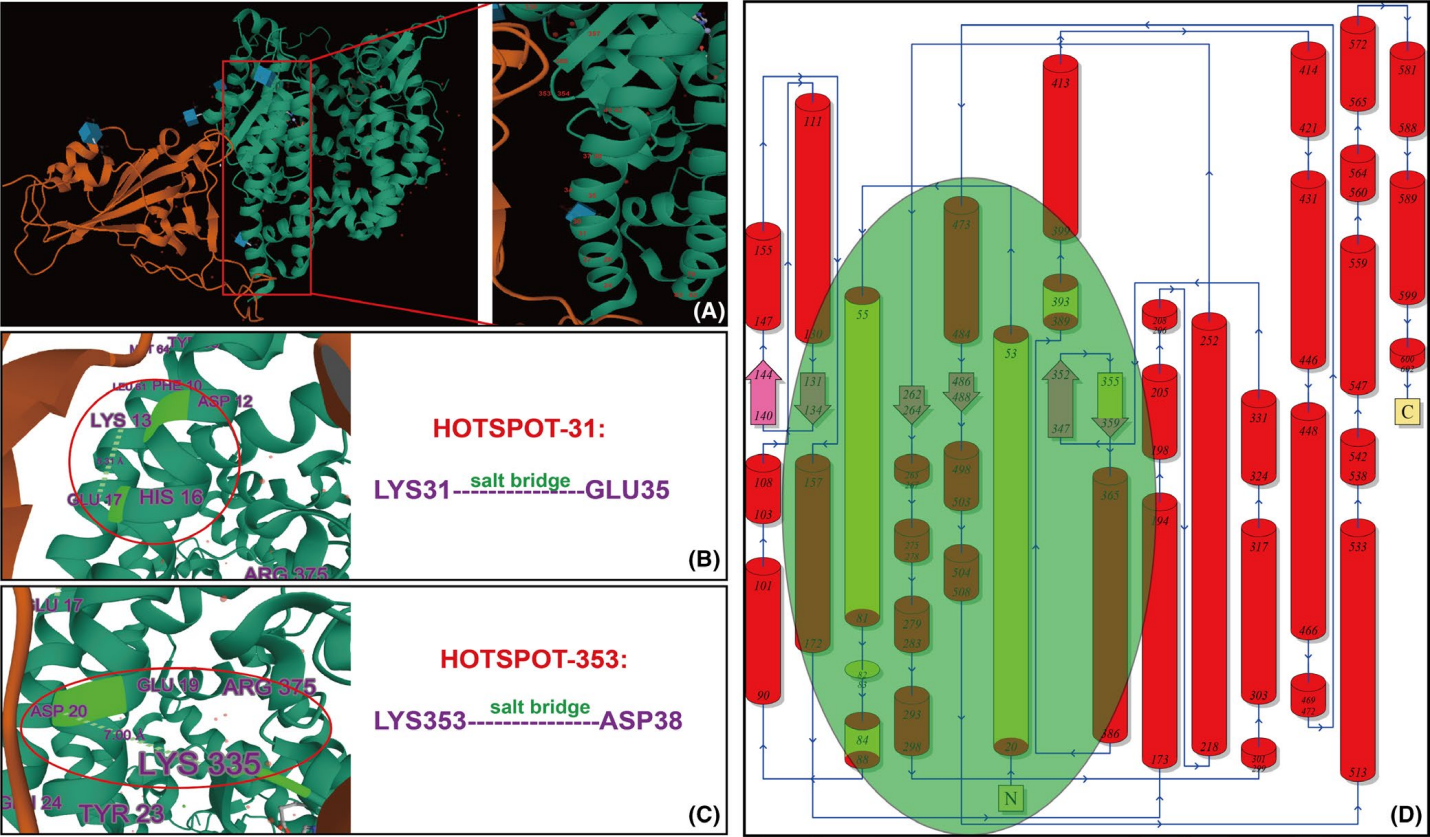
Schematic diagram of the combination of ACE2 and SARS‐CoV‐2‐RBD. (A) Three‐dimensional spatial distribution of 20 key amino acids of ACE2 binding to SARS‐CoV‐2‐RBD. The 20 amino acid sites are distributed on the same plane and arranged in a straight line; their spatial position is very close. It is suggested that we can design small‐molecule soluble ACE2 peptides combined with SARS‐CoV‐2‐RBD according to these 20 amino acid sites. (B, C) Three‐dimensional spatial diagram of two core regions of ACE2 and SARS‐CoV‐2‐RBD. (D) Schematic diagram of the distribution of ACE2 amino acids; the green area represents the key amino acids of ACE2 combined with SARS‐CoV‐2‐RBD. ACE2, angiotensin‐converting enzyme 2; SARS‐CoV‐2‐RBD, severe acute respiratory syndrome coronavirus 2 receptor binding domain

The recent discovery of the important binding of the SARS‐CoV‐2 spike protein to ACE2 provides researchers with important clues. There are two core regions involved in the binding of ACE2 protein to SARS‐CoV‐2‐RBD (Figure 1): (1) lysine 31, which is composed of lysine 31 forming a salt bridge with glutamate 35; and (2) lysine 353, which is composed of lysine 353 forming a salt bridge with aspartic acid 38.^28^ It was also found that the binding site of ACE2 for SARS‐CoV‐2‐RBD consists of 20 amino acids at the N‐terminal 24–393 of ACE2. These 20 amino acids are very similar in spatial structure and bind to 17 amino acids between 417 and 505 of SARS‐CoV‐2‐RBD, thereby allowing the entry of SARS‐CoV‐2 into the human body.^28^ Another study found that the binding of SARS‐CoV‐2‐RBD to ACE2 protein did not affect the biological activity and other physiological functions of the latter. In addition, some studies utilizing high‐throughput sequencing technology and flow cytometry have analyzed the effect of mutations in amino acid sites of the ACE2 protein on the binding capacity of ACE2 to SARS‐CoV‐2‐RBD. Moreover, data analysis studies have screened the key sites that can enhance SARS‐CoV‐2 infection. It was found that the binding ability of SARS‐CoV‐2‐RBD for ACE2 can be changed by point mutations of amino acids in the extracellular domain of the ACE2 protein. This provides valuable information for our research; after determining the structure of ACE2, each binding site is optimized to the maximum binding force by point mutation, and to find and confirm the best combination to resist the virus.

## 5 CONCLUSION

**FIGURE 2:**
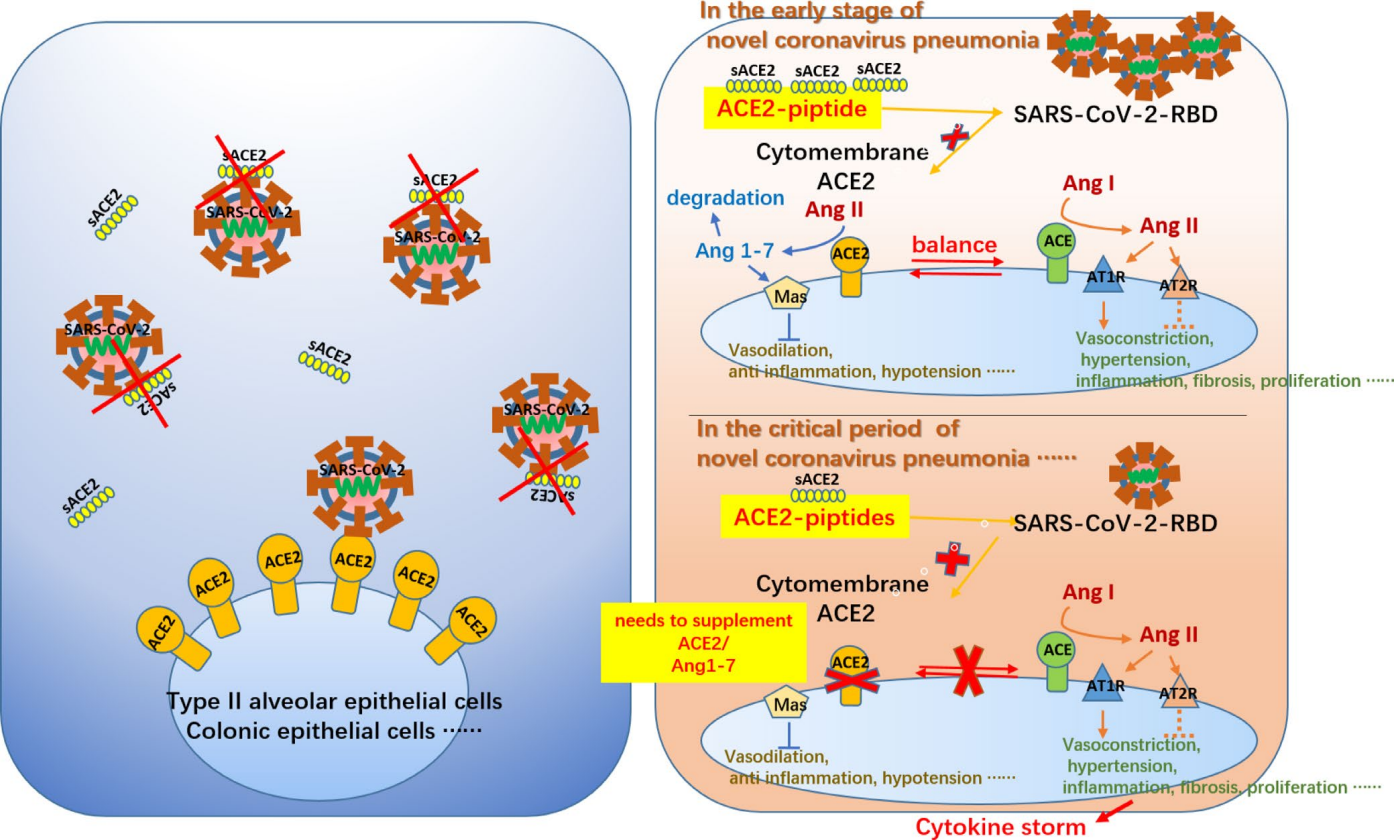
Schematic diagram of small‐molecule soluble ACE2 peptides used in the treatment of COVID‐19. ACE2, angiotensin‐converting enzyme 2; COVID‐19, coronavirus disease 2019

Therefore, we hypothesized that the appropriate treatment for SARS‐CoV‐2 infection should include drugs against the novel coronavirus combined with small‐molecule soluble ACE2 peptides. The latter can competitively bind SARS‐CoV‐2‐RBD via the extracellular domain of ACE2 on the cell membrane, reducing infection through blockage of viral entry into cells and inhibition of viral replication. As shown in Figure 2, patients with severe pneumonia are treated with anti‐novel coronavirus drugs combined with ACE2 peptides and angiotensin (1–7). Angiotensin (1–7) can compensate for acute injury caused by ACE2 deficiency in multiple organs. Furthermore, the inflammatory storm in patients with severe pneumonia occurs as a downstream event of acute lung injury. Using angiotensin (1–7) to suppress the inflammatory storm may help alleviate the symptoms of patients. This hypothesis also applies to patients with diabetes, hypertension, and chronic cardiovascular disease who are infected with SARS‐CoV‐2.

## CONFLICT OF INTEREST

The authors declare no competing financial interests.

## AUTHOR CONTRIBUTION

Y.L. and H.Z. searched and prepared the references. H.L. and Y.L. analyzed the data and wrote the manuscript.

## Data Availability

The data that support the findings of this study are available from the corresponding author upon reasonable request.
